# Repurposing Alone and in Combination of the Antiviral Saquinavir with 5-Fluorouracil in Prostate and Lung Cancer Cells

**DOI:** 10.3390/ijms232012240

**Published:** 2022-10-13

**Authors:** Mariana Pereira, Nuno Vale

**Affiliations:** 1 OncoPharma Research Group, Center for Health Technology and Services Research (CINTESIS), Rua Doutor Plácido da Costa, s/n, 4200-450 Porto, Portugal; 2 Institute of Biomedical Sciences Abel Salazar (ICBAS), University of Porto, Rua Jorge Viterbo Ferreira, 228, 4050-313 Porto, Portugal; 3 CINTESIS@RISE, Faculty of Medicine, University of Porto, Alameda Professor Hernâni Monteiro, 4200-319 Porto, Portugal; 4 Department of Community Medicine, Information and Health Decision Sciences (MEDCIDS), Faculty of Medicine, University of Porto, Rua Doutor Plácido da Costa, s/n, 4200-450 Porto, Portugal

**Keywords:** saquinavir, 5-fluorouracil, drug repurposing, drug combination, cancer

## Abstract

Prostate and lung cancers are among the most common cancer types, and they still need more therapeutics. For this purpose, saquinavir (SAQ) was tested alone and in combination with 5-fluorouracil (5-FU). PC-3 and A549 cells were exposed to increasing concentrations of both drugs alone or in combination, with simultaneous or sequential administration. Cell viability was obtained using the MTT assay and synergism values using CompuSyn software. Results showed that SAQ was the more cytotoxic of both drugs in PC-3 cells, while 5-FU was the most cytotoxic in A549 cells. When these drugs were used in combination, the more synergistic combination in PC-3 cells was the IC_50_ of SAQ with various concentrations of 5-FU, particularly when 5-FU was only applied 24 h later. Meanwhile for A549 the most promising combination was 5-FU with delayed SAQ, but with a weaker effect than all combinations demonstrated in PC-3 cells. These results demonstrate that SAQ could be used as a new repurposed drug for the treatment of prostate cancer and this treatment potential could be even greater if SAQ is combined with the anticancer drug 5-FU, while for lung cancer it is not as efficient and, therefore, not of as much interest.

## 1. Introduction

Prostate cancer is the second most common cancer among men worldwide, only behind skin cancer, with almost 1.5 million cases diagnosed in 2020 alone [1]. Survival rates depend on where the cancer is found, with higher rates when it is localized in the prostate area than after it spreads to other parts of the body, decreasing from a near 100% 5-year survival rate to 32.3% [2]. This is a very heterogeneous cancer with multiple classifications, such as high or low grade and aggressive or non-aggressive, with most cases occurring at later ages (55 and above) [3]. Geography, ethnicity, and race are factors that vary prostate cancer rates, with higher numbers in African-descendant men, and it is also the cancer type with the highest inheritability [4]. Lung cancer is among the leading causes of cancer associated deaths and is associated with risk factors such as smoking and air pollution [5]. Globally, there has been an increase in lung cancer incidence, and, while this cancer type affects more men than women, there has been an increase in cases affecting the latter [6]. With more than 2.2 million new cases and 1.7 million deaths in 2020 alone, lung cancer is a concern that urgently requires research for better treatments [7]. 

Treatments are still needed for these diseases, and an emerging technology has been drug repurposing, which entails the administration of drugs that have previously received approval but for a new indication. This has various benefits, such that the drug has previously been shown to be suitable for human administration, that the drug research process is sped up, and that it also costs less [8].

Saquinavir (SAQ) was the first protease inhibitor developed for the treatment of HIV, introduced into the market in 1995 under the name Invirase (Roche Laboratories Inc). This drug acts as a peptide-like substrate analog that binds to the active site of the HIV protease and inhibits it [9]. This causes the inhibition of the cleavage of the *gal-pro* fusion protein, important for virus maturation, leading to the development of immature and structurally defective viral particles. SAQ has low bioavailability and is metabolized mainly by the CYP3A4 of the cytochrome P450 in the liver, which is normally administered to patients in combination with a CYP inhibitor, such as ritonavir [10].

Several studies have tested SAQ in numerous types of cancer cells, from Kaposi sarcoma to cervical cancer and bladder cancer [11,12,13], among others, and it has shown to be effective in decreasing cancer cell proliferation [13,14,15,16], and causing proteotoxic stress [16], anti-tumor growth [11], apoptosis [14,17], anti-angiogenesis [11], and increased radiosensitization [17,18], alone or in combination with other drugs [14,15,16]. With this in mind, the present study aimed to assess the cytotoxic effect of SAQ alone and in combination with 5-fluorouracil (5-FU), a reference drug used to treat multiple types of cancer, in prostate and lung cancer cells.

## 2. Results

### 2.1. PC-3 Cells

#### 2.1.1. Cytotoxicity of 5-FU

**Figure 1:**
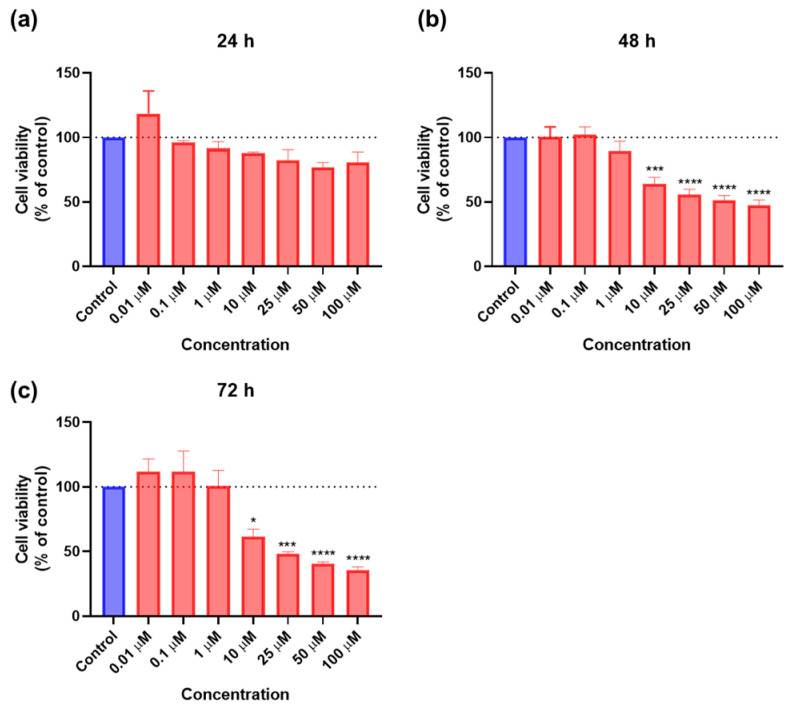
Cytotoxic results of PC-3 after exposure to increasing concentrations of 5-FU (0.01–100 μM) for 24 h (**a**), 48 h (**b**), and 72 h (**c**). Control cells were treated with 0.01% DMSO (vehicle). Cell viability was obtained using the MTT assay and the results are given as the mean ± SEM (24 h *n* = 3, 48 h, and 72 h *n* = 6). * Statistically significant vs. control (vehicle) at *p* < 0.05; *** statistically significant vs. control (vehicle) at *p* < 0.001; **** statistically significant vs. control (vehicle) at *p* < 0.0001.

**Figure 2:**
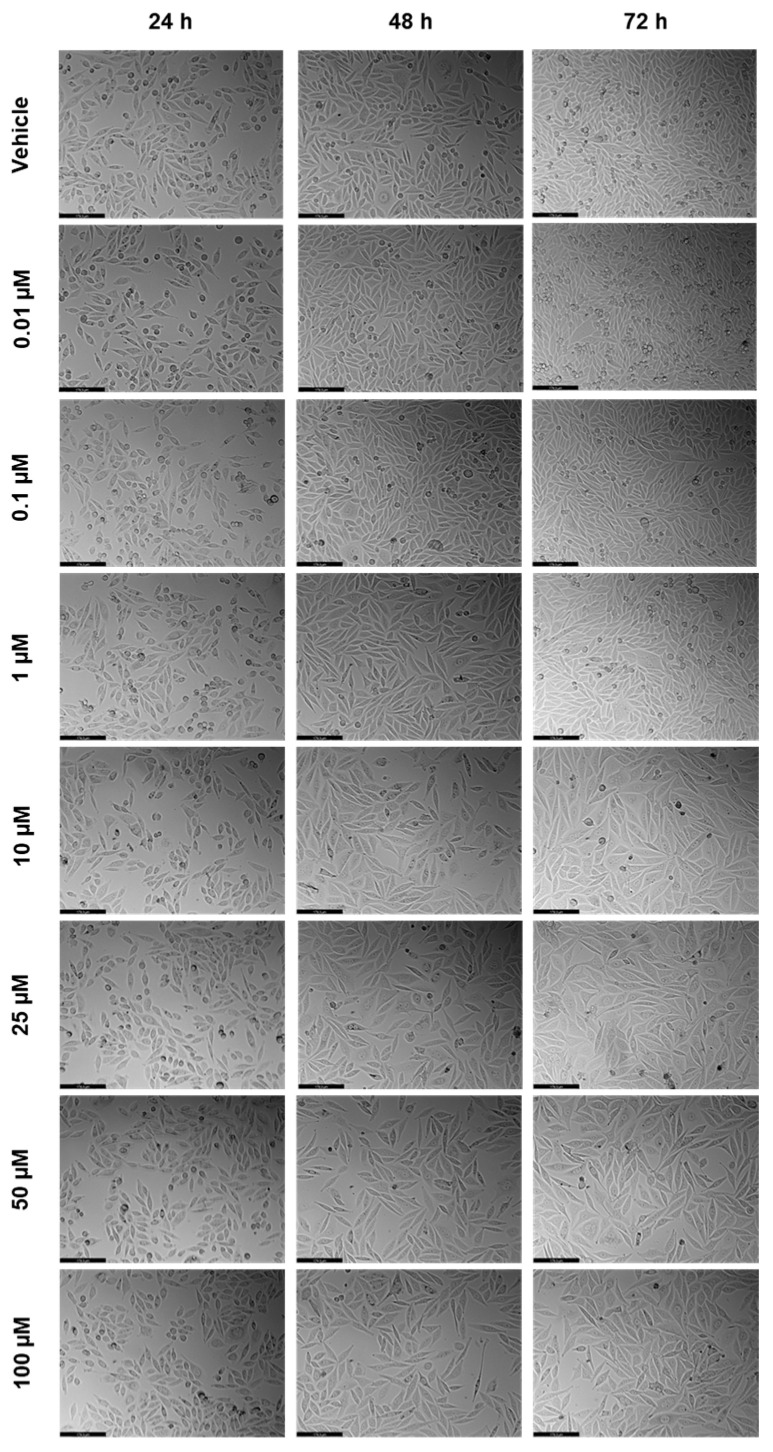
Morphological evaluation of PC-3 after exposure to increasing concentrations of 5-FU (0.01–100 μM) for 24, 48, and 72 h. Control cells were treated with the vehicle (0.01% DMSO). These images are representative of three independent experiments for 24 h, and six independent experiments for 48 h and 72 h. The scale bar is 200 μM.

As a reference drug, 5-FU is used to treat multiple types of cancer, such as breast and colorectal cancer [19]. Since this is the case, this drug was used and tested in the PC-3 prostate cancer cells used in this study, at concentrations of 0.01, 0.1, 1, 10, 25, 50, and 100 μM at three time points (24 h, 48 h, and 72 h). The cell viability results and the morphological evaluation are represented in Figure 1 and Figure 2, respectively. The 5-FU drug had almost no effect during 24 h, only starting to significantly decrease cell viability at 48 h. This decrease was time-dependent, with it being greater at 72 h, and it was also concentration-dependent, with the first concentration that had any effect being 10 μM and only reaching 50% inhibition values after 25 μM (Figure 1). 

**Figure 3:**
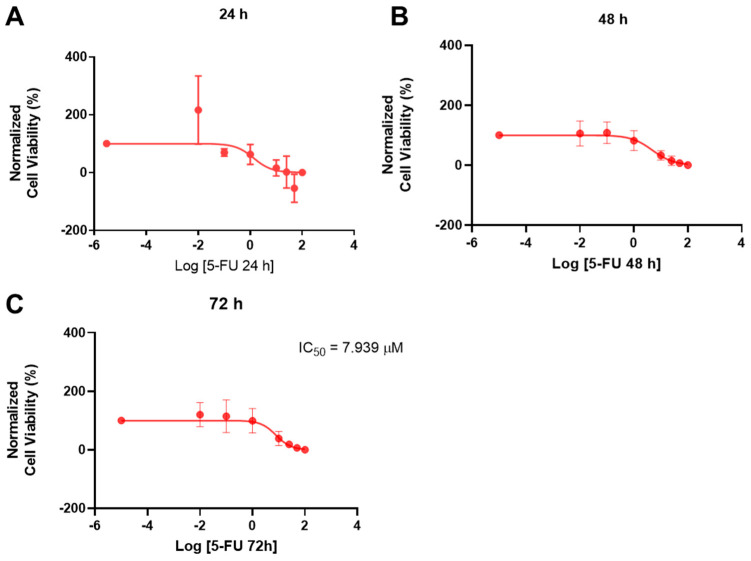
Dose-response curve and IC50 of PC-3 after exposure to increasing concentrations of 5-FU (0.01−100 μM) for 24 h (**A**), 48 h (**B**), and 72 h (**C**). Control cells were treated with 0.01% DMSO (vehicle). Cell viability was obtained using the MTT assay and the results were normalized and are given as the mean ± SEM (24 h *n* = 3, 48 h. and 72 h *n* = 6).

When constructing the dose-response curves, as expected, no IC_50_ values were obtained for 24 h and 48 h, and the value obtained for 72 h (7.939 μM) was too low when considering the results obtained in the bar graph of cell viability (Figure 3), where there was a pronounced decrease related to 50% inhibition only with concentrations above 25 μM of 5-FU. This demonstrates that, although 5-FU did not have the same activity in prostate cancer that it has been proved to have in other cancer types, it can cause cytotoxicity to these cells at higher concentrations.

#### 2.1.2. Cytotoxicity of SAQ

**Figure 4:**
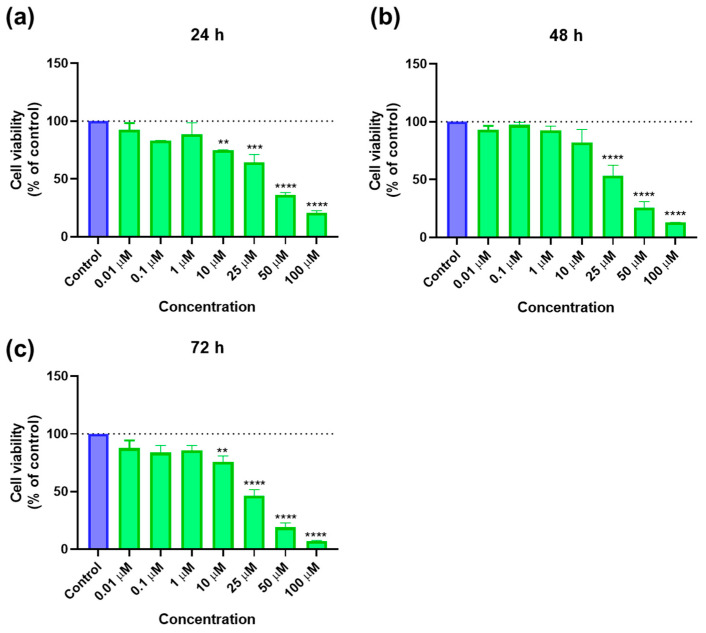
Cytotoxic results of PC-3 after exposure to increasing concentrations of saquinavir (0.01–100 μM) for 24 h (**a**), 48 h (**b**), and 72 h (**c**). Control cells were treated with 0.01% DMSO (vehicle). Cell viability was obtained using the MTT assay and the results are given as the mean ± SEM (24 h *n* = 3, 48 h, and 72 h *n* = 6). ** statistically significant vs. control (vehicle) at *p* < 0.01; *** statistically significant vs. control (vehicle) at *p* < 0.001; **** statistically significant vs. control (vehicle) at *p* < 0.0001.

**Figure 5:**
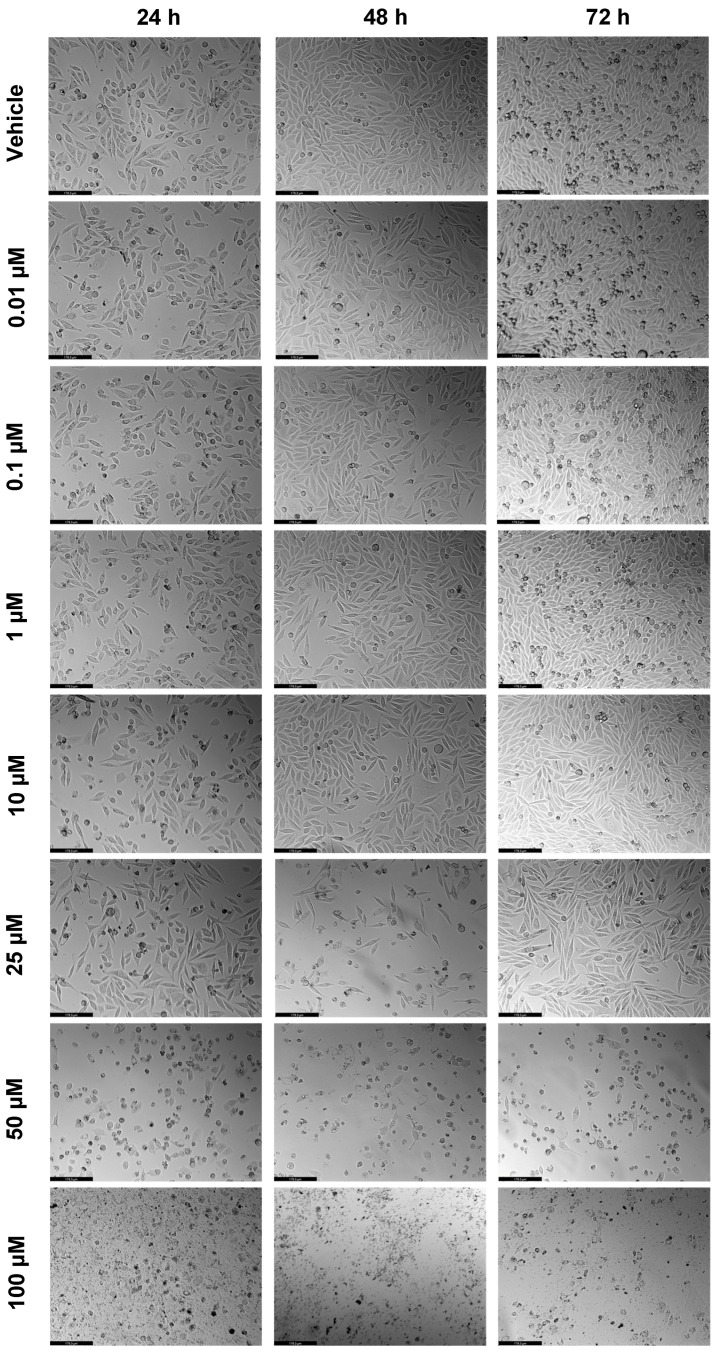
Morphological evaluation of PC-3 after exposure to increasing concentrations of SAQ (0.01–100 μM) for 24, 48, and 72 h. Control cells were treated with the vehicle (0.01% DMSO). These images are representative of three independent experiments for 24 h, and six independent experiments for 48 h and 72 h. The scale bar is 200 μM.

In this study, SAQ was tested alone in PC-3 cells to understand its cytotoxicity, using the same concentrations (0.01, 0.1, 1, 10, 25, 50, and 100 μM) and time points (24 h, 48 h, and 72 h) as the ones tested with 5-FU. The results of cell viability and morphologic evaluation are represented in Figure 4 and Figure 5, respectively. SAQ caused a decrease of total cells at all time points, significantly after 10 μM, a concentration-dependent decrease (Figure 4). Variations in cell viability with time were minimal, which could point to SAQ having a strong effect right after administration but not after some time.

**Figure 6:**
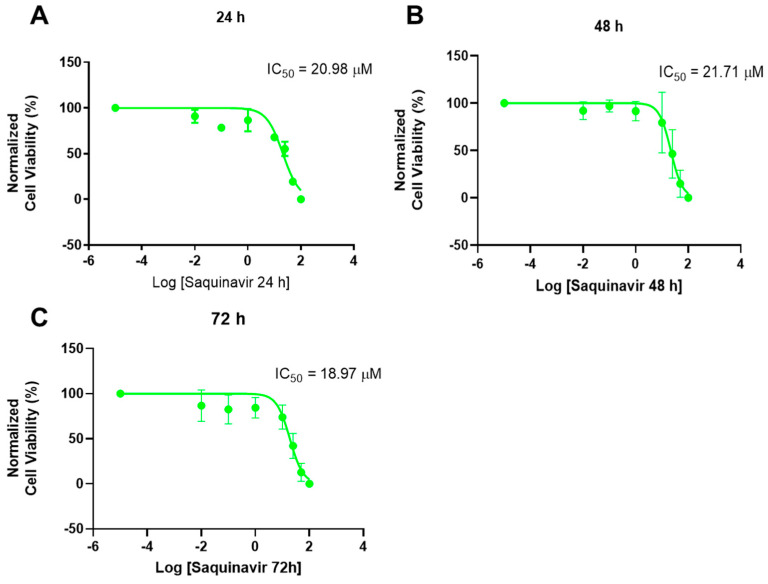
Dose-response curve and IC_50_ of PC-3 after exposure to increasing concentrations of saquinavir (0.01−100 μM) for 24 h (**A**), 48 h (**B**), and 72 h (**C**). Control cells were treated with 0.01% DMSO (vehicle). Cell viability was obtained using the MTT assay and the results were normalized and are given as the mean ± SEM (24 h *n* = 3, 48 h, and 72 h *n* = 6).

Contrary to what was obtained with 5-FU, the dose-response curves of SAQ gave better results, with IC_50_ of 20.98 μM, 21.71 μM, and 18.97 μM after 24 h, 48 h, and 72 h, respectively. These similar IC_50_ values, despite more time being given for SAQ activity on the cells, give further evidence that the cytotoxic effect of SAQ mostly occurs in the first 24 h, plateauing after that (Figure 6). 

The IC_50_ of all the times and drugs is summarized in Table 1. As previously mentioned, no IC_50_ was obtained for 5-FU at 24 h and 48 h, only showing a stronger effect after 72 h.

#### 2.1.3. Combination Studies

##### Simultaneous Drug Addition

**Figure 7:**
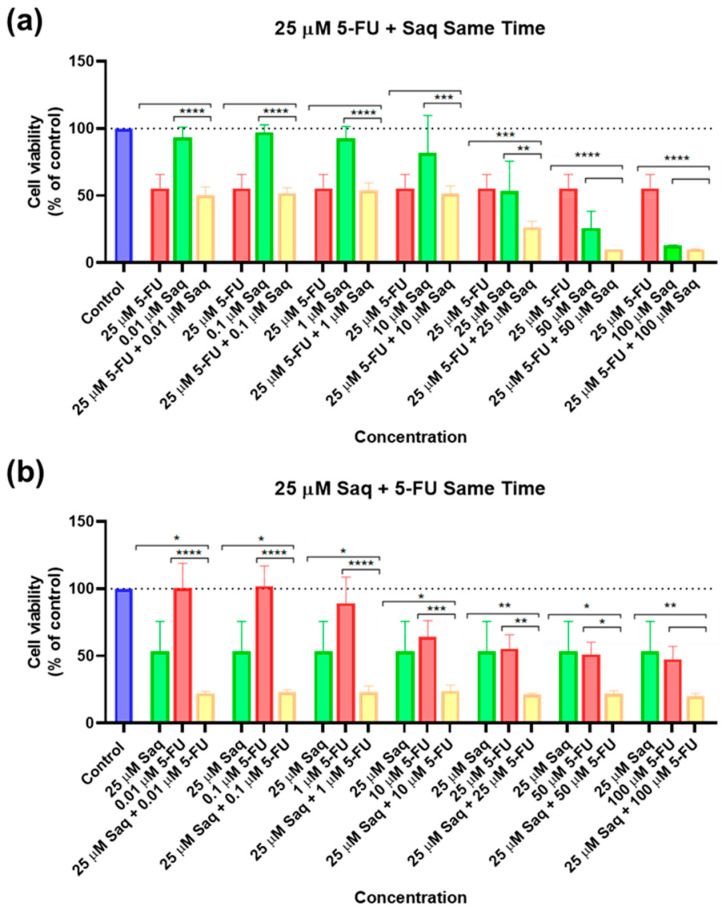
Cytotoxic results of PC-3 after exposure to drug combinations at the same time for 48 h. (**a**) Combination of 25 μM of 5-FU with increasing concentrations of saquinavir (0.01–100 μM); (**b**) combination of 25 μM of saquinavir with increasing concentrations of 5-FU (0.01–100 μM). Control cells were treated with 0.01% DMSO (vehicle). Cell viability was obtained using the MTT assay and the results are given as the mean ± SEM (*n* = 3). * Statistically significant vs. drug alone at *p* < 0.05; ** statistically significant vs. drug alone at *p* < 0.01; *** statistically significant vs. drug alone at *p* < 0.001; **** statistically significant vs. drug alone at *p* < 0.0001.

**Figure 8:**
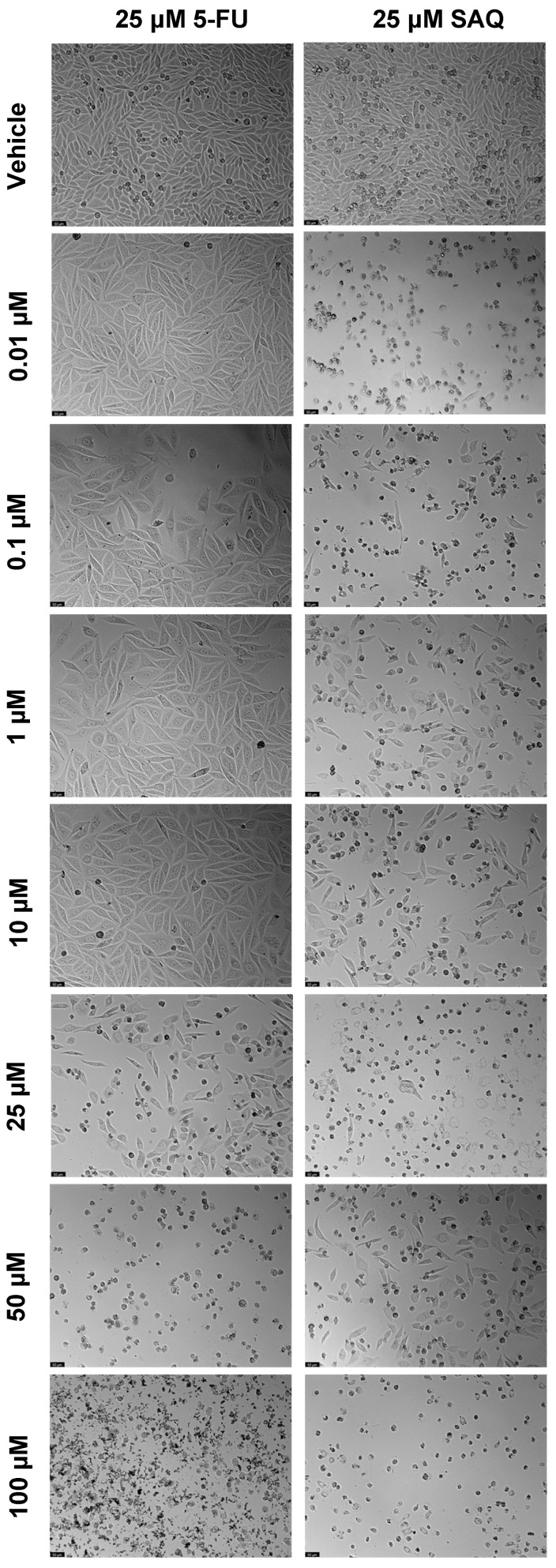
Morphological evaluation of PC-3 after exposure to combinations of 25 μM of 5-FU with increasing concentrations of saquinavir (0.01–100 μM) or 25 μM of saquinavir with increasing concentrations of 5-FU (0.01–100 μM) for 48 h. Both drugs were added at the same time. Control cells were treated with the vehicle (0.01% DMSO). These images are representative of three independent experiments. The scale bar is 200 μM.

After testing the drugs alone and obtaining an IC_50_ for SAQ, the next step was testing combinations of these two drugs. Furthermore, 25 μM of 5-FU and 25 μM of SAQ were chosen, as they are close to the IC_50_ of SAQ and of the concentration of 5-FU that started having a 50% inhibitory effect. Each of these concentrations was combined with 0.01, 0.1, 1, 10, 25, 50, and 100 μM of the other drug. Firstly, the drug combination was tested with drugs added at the same time. The results of cell viability and morphological evaluation are shown in Figure 7 and Figure 8, respectively. The combination of increasing SAQ concentrations to a fixed 25 μM of 5-FU had a stronger impact on cell viability variability (Figure 7a) than the combination of increasing 5-FU concentrations with a fixed 25 μM SAQ concentration (Figure 7b) when the drugs are added at the same time. Cell viability decreased in an SAQ concentration-dependent manner with the fixed 5-FU (Figure 7a), but when the 5-FU concentration was varied and combined with a fixed SAQ concentration there was practically no change in the combination cell viability values no matter how high the 5-FU concentration was, even if this decrease was significantly lower in comparison with the other combination, which points to the conclusion that SAQ was responsible for much of the effect (Figure 7b).

##### Sequential Drug Administration

**Figure 9:**
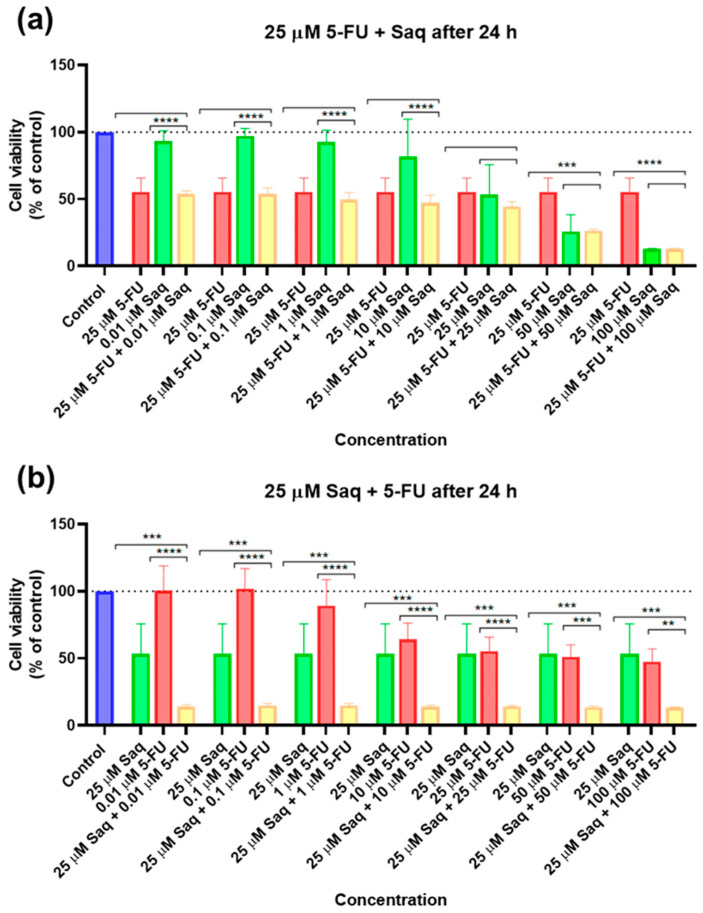
Cytotoxic results of PC-3 after exposure to drug combinations with the second drug only being added 24 h after the first. (**a**) Combination of 25 μM of 5-FU with increasing concentrations of saquinavir (0.01–100 μM); (**b**) combination of 25 μM of saquinavir with increasing concentrations of 5-FU (0.01–100 μM). Control cells were treated with 0.01% DMSO (vehicle). Cell viability was obtained using the MTT assay after 48 h, and the results are given as the mean ± SEM (*n* = 3). ** statistically significant vs. drug alone at *p* < 0.01; *** statistically significant vs. drug alone at *p* < 0.001; **** statistically significant vs. drug alone at *p* < 0.0001.

**Figure 10:**
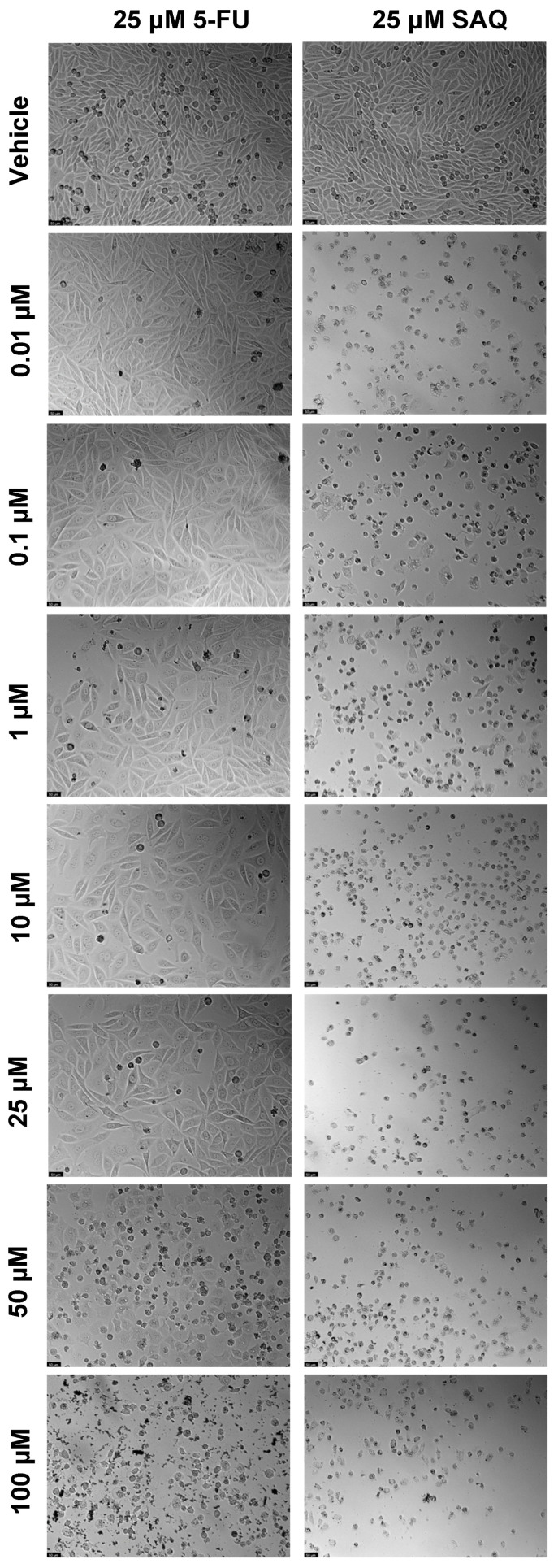
Morphological evaluation of PC-3 after exposure to combination of 25 μM of 5-FU with increasing concentrations of saquinavir (0.01–100 μM) or 25 μM of saquinavir with increasing concentrations of 5-FU (0.01–100 μM) for 48 h. The second drug was only added 24 h after the first drug. Control cells were treated with the vehicle (0.01% DMSO). These images are representative of three independent experiments. The scale bar is 200 μM.

The results of cell viability and morphological evaluation of drug combinations where the second drug was added 24 h after the fixed drug, are shown in Figure 9 and Figure 10, respectively. The addition of increasing SAQ concentrations 24 h after exposure to 25 μM of 5-FU led to a decrease in cell viability in a concentration-dependent manner (Figure 9a), although to a lesser extent than when both drugs were added to cells at the same time (Figure 7a). This decrease also only started being significant at 50 μM, while when added at the same time this was already noticed at 25 μM. Again, the posterior addition of increasing concentrations of 5-FU to 25 μM of SAQ had a greater impact on cell viability that was not dependent on 5-FU concentration (Figure 9b). This effect was, however, stronger than when both drugs are added simultaneously (Figure 7b).

##### Synergism Results

The cell viability of drug combinations was used to perform a synergy study using CompuSyn software. The results obtained for the effect of the drug combination and for the combination index (CI) can be seen in Table 2. The fractional effect (Fa) denotes cellular death and ranges from 0, which is no cellular death due to no effect of the drug combination, to 1, which is complete cellular death.

Most of these results are indicative of synergism. When looking at the values, and since the lower the values are the more synergic the combination is, it can be seen that combining 25 μM of 5-FU with increasing concentrations of SAQ works better when both drugs are added at the same time than 24 h apart, particularly after a concentration of 25 μM of SAQ. Meanwhile, when looking at the combination of 25 μM of SAQ with increasing concentrations of 5-FU, the values of the combination are better when 5-FU is added only 24 h after SAQ, although the synergism of the combination decreases slightly with high concentrations of 5-FU. Overall, the better combination seems to be 25 μM of SAQ with various concentrations of 5-FU, with the latter being administrated 24 h after SAQ, as the cell viability results also pointed out. 

### 2.2. A549 Cells

#### 2.2.1. Cytotoxicity of 5-FU

**Figure 11:**
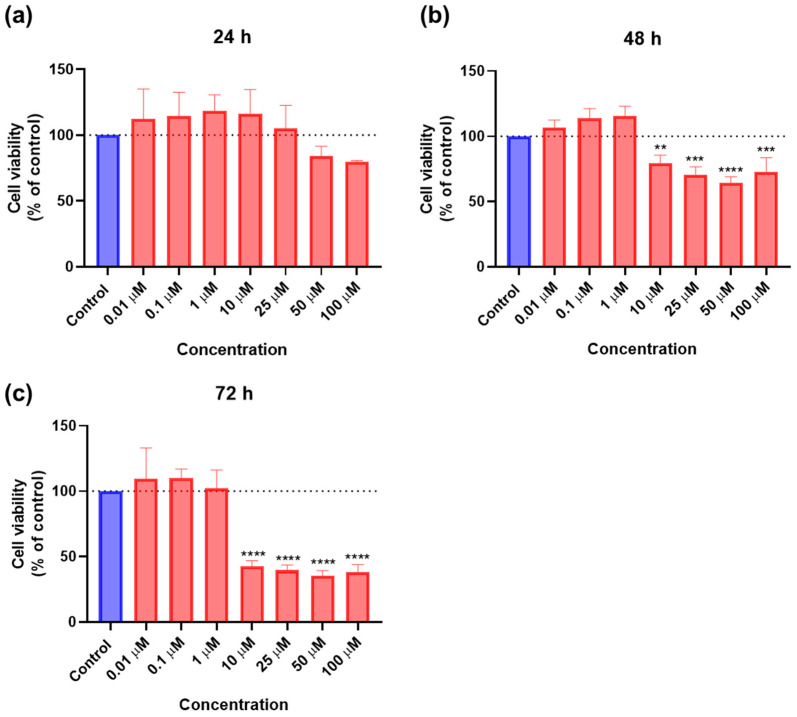
Cytotoxic results of A549 after exposure to increasing concentrations of 5-FU (0.01–100 μM) for 24 h (**a**), 48 h (**b**), and 72 h (**c**). Control cells were treated with 0.01% DMSO (vehicle). Cell viability was obtained using the MTT assay and the results are given as the mean ± SEM (*n* = 3). ** Statistically significant vs. control (vehicle) at *p* < 0.01; *** statistically significant vs. control (vehicle) at *p* < 0.001; **** statistically significant vs. control (vehicle) at *p* < 0.0001.

**Figure 12:**
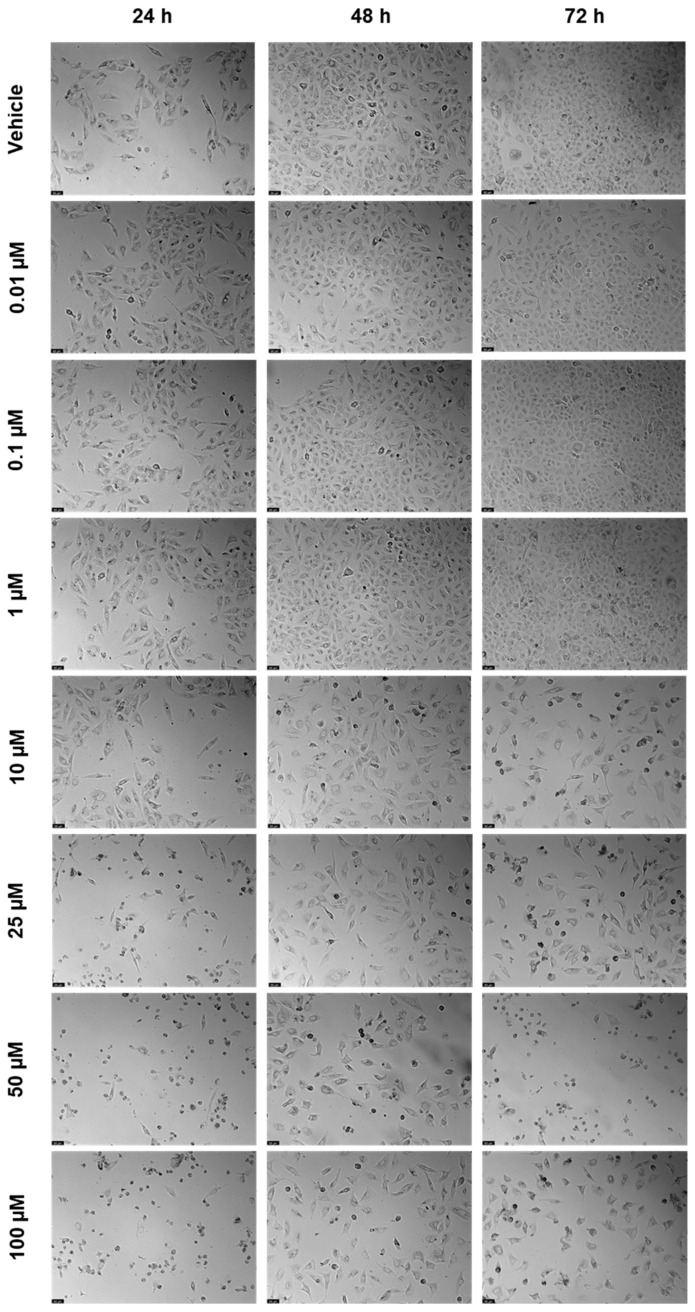
Morphological evaluation of A549 after exposure to increasing concentrations of 5-FU (0.01–100 μM) for 24, 48, and 72 h. Control cells were treated with the vehicle (0.01% DMSO). These images are representative of three independent experiments. The scale bar is 200 μM.

After obtaining promising results using PC-3 cells, there was an interest in performing the same experiments in another cancer cell line, in order to understand if these results could be reproduced. A549 lung carcinoma epithelial cells were chosen for this, and, firstly, the cytotoxicity of 5-FU was tested for concentrations of 0.01, 0.1, 1, 10, 25, 50, and 100 μM at three time points (24 h, 48 h, and 72 h). The cell viability results and the morphological evaluation are represented in Figure 11 and Figure 12, respectively. While at 24 h there was no significant effect on cell viability, at 48 and 72 h there were marked effects for concentrations of 10 μM and above, which indicates a time-dependent effect of this drug. However, this effect was not concentration-dependent, especially at 72 h, indicating a maximum concentration that is effective in these cells. At 48 h there was even a slight increase in cell viability for the highest concentration.

**Figure 13:**
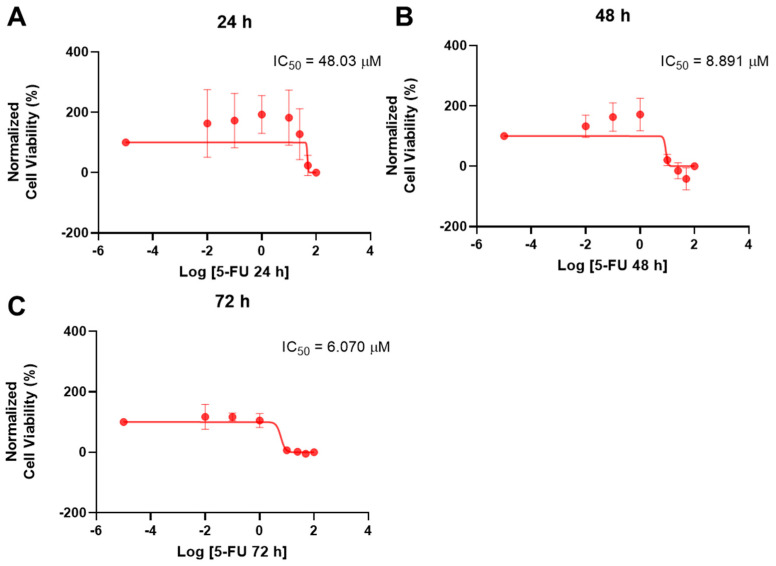
Dose-response curve and IC_50_ of A549 after exposure to increasing concentrations of 5-FU (0.01–100 μM) for 24 h (**A**), 48 h (**B**), and 72 h (**C**). Control cells were treated with 0.01% DMSO (vehicle). Cell viability was obtained using the MTT assay and the results were normalized and are given as the mean ± SEM (*n* = 3).

When looking at the dose-response curves and IC_50_ values calculated from the cell viability results (Figure 13), there is, as expected, a significant decrease in IC_50_ from almost 50 μM at 24 h to 9 and 6 μM for 48 and 72 h, respectively.

#### 2.2.2. Cytotoxicity of SAQ

**Figure 14:**
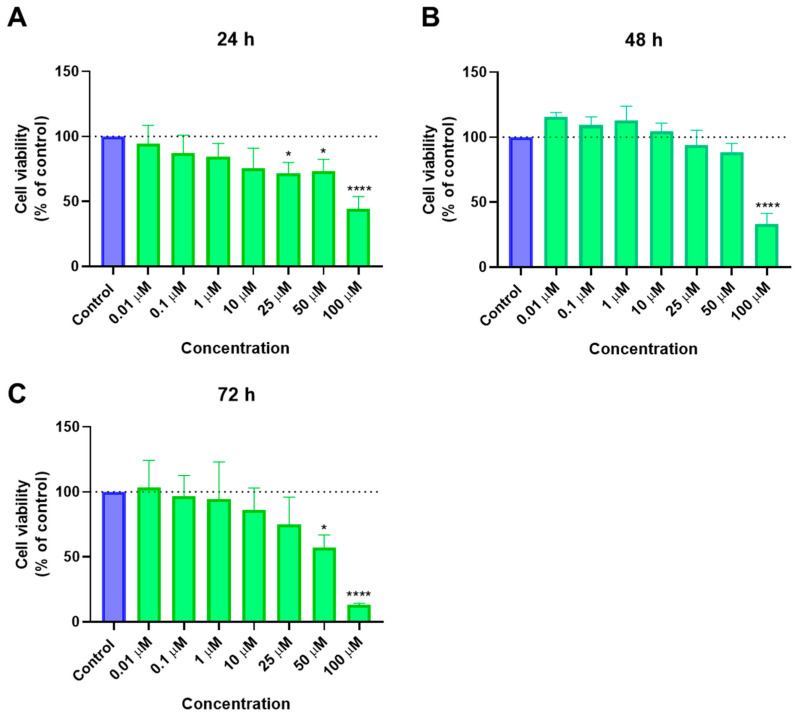
Cytotoxic results of A549 after exposure to increasing concentrations of saquinavir (0.01–100 μM) for 24 h (**A**), 48 h (**B**), and 72 h (**C**). Control cells were treated with 0.01% DMSO (vehicle). Cell viability was obtained using the MTT assay and the results are given as the mean ± SEM (*n* = 3). * Statistically significant vs. control (vehicle) at *p* < 0.05; **** statistically significant vs. control (vehicle) at *p* < 0.0001.

**Figure 15:**
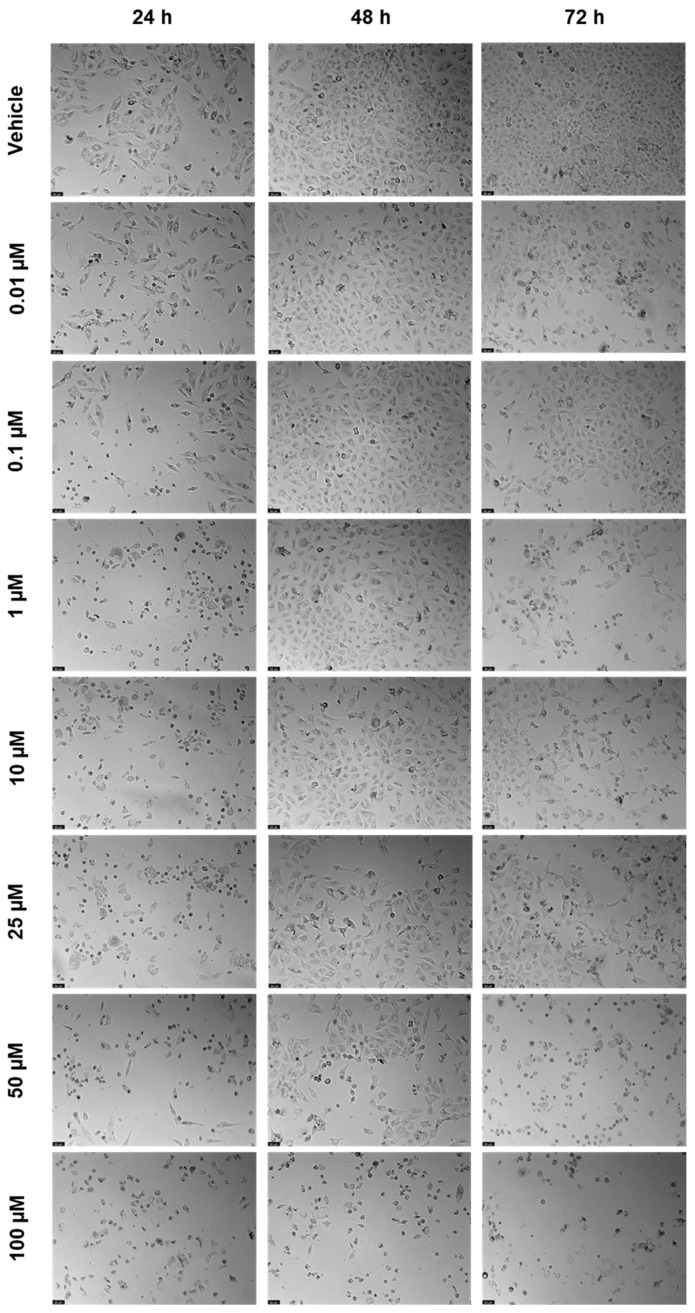
Morphological evaluation of A549 after exposure to increasing concentrations of saquinavir (0.01–100 μM) for 24, 48, and 72 h. Control cells were treated with the vehicle (0.01% DMSO). These images are representative of three independent experiments. The scale bar is 200 μM.

SAQ was tested alone in A549 cells to understand its cytotoxicity, using the same concentrations (0.01, 0.1, 1, 10, 25, 50, and 100 μM) and time points (24 h, 48 h, and 72 h) as the ones tested for 5-FU. The results of cell viability and morphologic evaluation are represented in Figure 14 and Figure 15, respectively. There was a slight decrease in cell viability at 24 h for concentrations of 25 and 50 μM, as well as a more marked decrease for 100 μM (Figure 14A). However, for 48 h, the cell viability was only significantly affected for concentrations of 100 μM (Figure 14B), and at 72 h for 50 and 100 μM, with a higher effect (Figure 14C). The cytotoxicity was, therefore, concentration-dependent for all time points, but there was a lower effect when exposed for 48 h than for 24 h and 72 h. 

**Figure 16:**
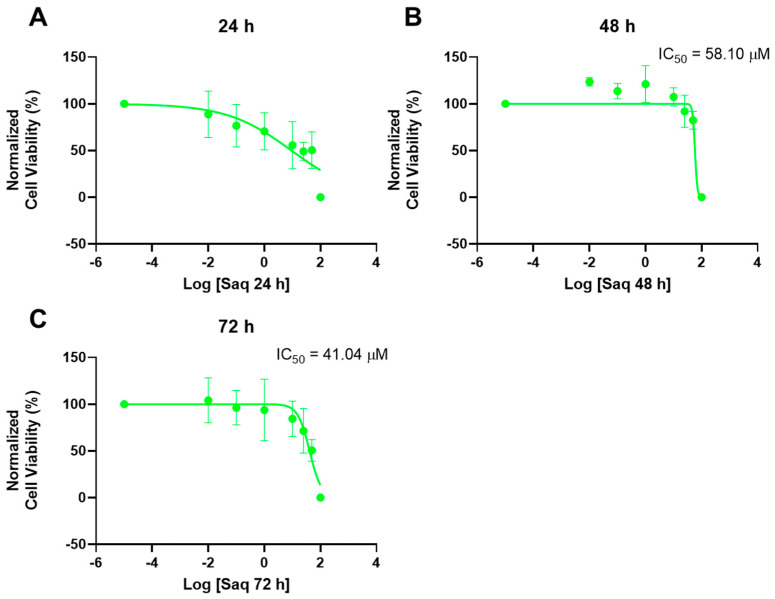
Dose-response curve and IC_50_ of A549 after exposure to increasing concentrations of saquinavir (0.01−100 μM) for 24 h (**A**), 48 h (**B**), and 72 h (**C**). Control cells were treated with 0.01% DMSO (vehicle). Cell viability was obtained using the MTT assay and the results were normalized and are given as the mean ± SEM (*n* = 3).

In Figure 16 the dose-response curves and IC_50_ of saquinavir can be seen. Despite this drug having significant cytotoxicity at 24 h, no IC_50_ was able to be calculated. Meanwhile, IC_50_ values of 58.10 μM and 41.04 μM for 48 and 72 h were obtained. These values were as per the cell viability graphs in Figure 15.

When comparing the IC_50_ values of 5-FU and SAQ in A549 cells (Table 3) it is clear that 5-FU has a stronger effect on cell viability than SAQ. At 24 h, no IC_50_ was obtained for SAQ, while for 5-FU it was possible, even if it is a high value of 48.03 μM. Both drugs decreased the IC_50_ values with time, with values for 5-FU considerably lower than the SAQ ones.

All IC_50_ values calculated in this study are summarized in Table 4. While for PC-3 cells SAQ was more cytotoxic, in A549 it is 5-FU that is more effective, with values lower than those calculated for PC-3 for 48 and 72 h. SAQ is less effective on A549 cells than on PC-3 cells, needing higher concentrations to have the same effect.

#### 2.2.3. Combination Studies

##### Simultaneous Drug Addition

**Figure 17:**
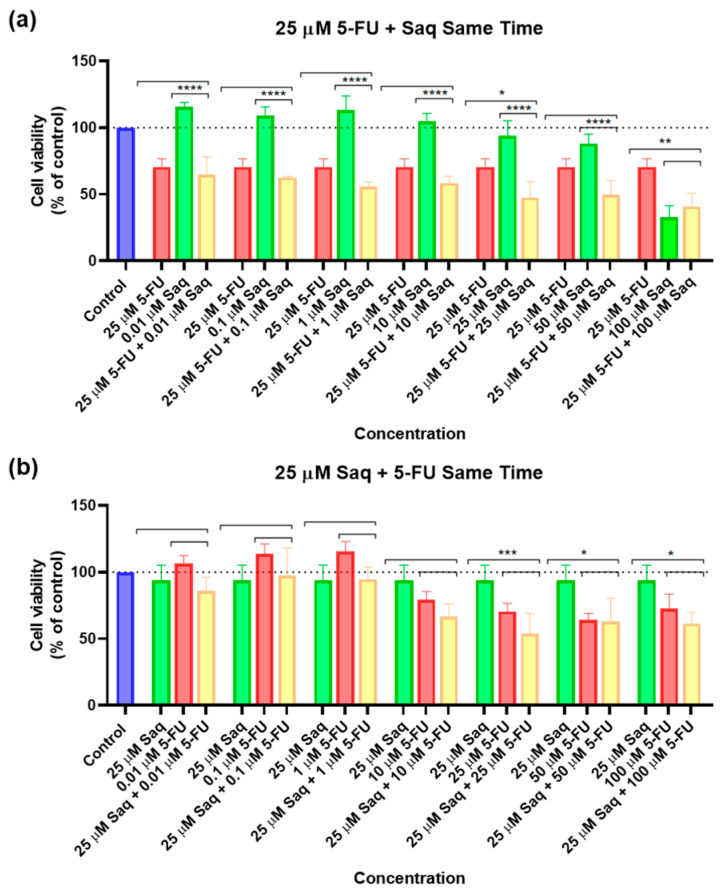
Cytotoxic results of A549 after exposure to drug combinations at the same time for 48 h. (**a**) Combination of 25 μM of 5-FU with increasing concentrations of saquinavir (0.01–100 μM); (**b**) combination of 25 μM of saquinavir with increasing concentrations of 5-FU (0.01–100 μM). Control cells were treated with 0.01% DMSO (vehicle). Cell viability was obtained using the MTT assay and the results are given as the mean ± SEM (*n* = 3). * Statistically significant vs. drug alone at *p* < 0.05; ** statistically significant vs. drug alone at *p* < 0.01; *** statistically significant vs. drug alone at *p* < 0.001; **** statistically significant vs. drug alone at *p* < 0.0001.

**Figure 18:**
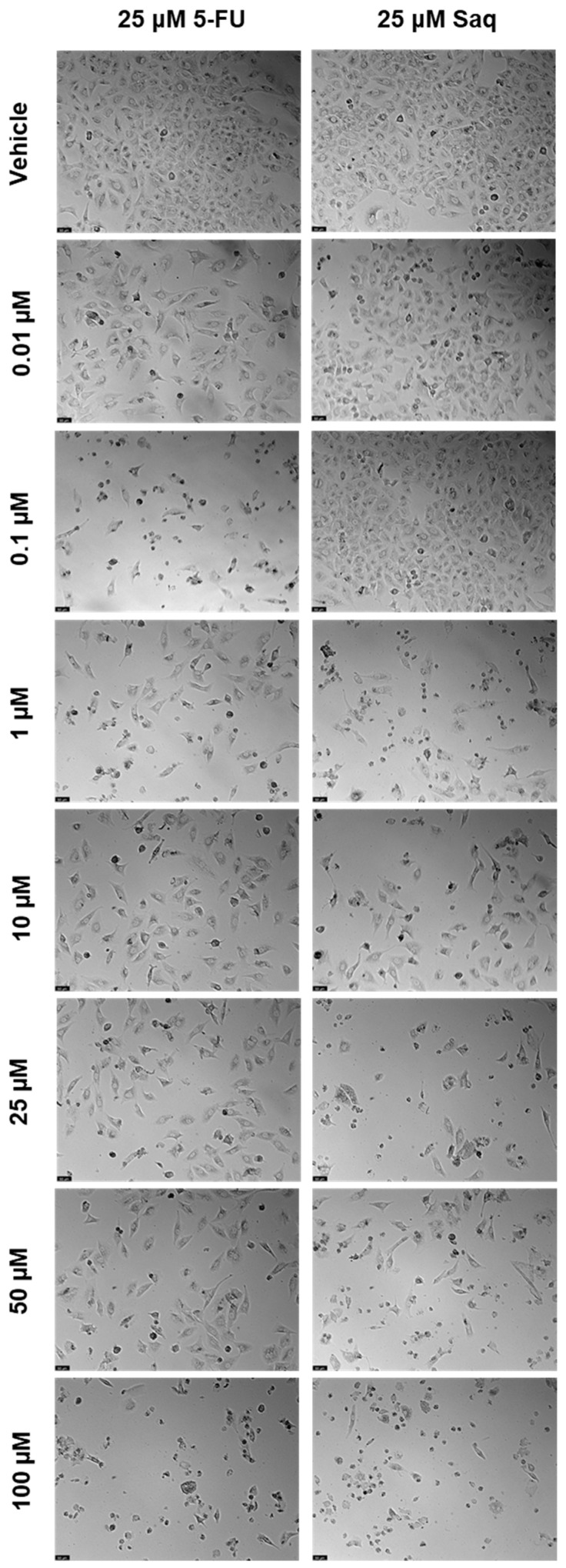
Morphological evaluation of A549 after exposure to combinations of 25 μM of 5-FU with increasing concentrations of saquinavir (0.01–100 μM) or 25 μM of saquinavir with increasing concentrations of 5-FU (0.01–100 μM) for 48 h. Both drugs were added at the same time. Control cells were treated with the vehicle (0.01% DMSO). These images are representative of three independent experiments. The scale bar is 200 μM.

Furthermore, 25 μM of 5-FU and 25 μM of SAQ were combined with 0.01, 0.1, 1, 10, 25, 50, and 100 μM of the other drug. Firstly, the drug combination was tested with drugs added at the same time. The results of cell viability and morphological evaluation are shown in Figure 17 and Figure 18, respectively. The combination of 25 μM of 5-FU with increasing concentrations of SAQ proved to be better than using SAQ alone, but the majority of cytotoxicity seems to be related to 5-FU (Figure 17a). The only decrease in cell viability that is statistically significant from 5-FU and SAQ alone is for 25 μM of 5-FU combined with 25 μM of SAQ. Meanwhile, for the combinations of 25 μM SAQ with increasing 5-FU concentrations, the combinations were never better than both the drugs alone, with combinations only being more effective for 25, 50, and 100 μM of 5-FU with 25 μM of SAQ compared with 25 μM of SAQ alone (Figure 17b). Overall, the combination of 25 μM of 5-FU with increasing concentrations of SAQ at the same time had a higher effect on cell viability than the opposite, which is expected since 5-FU was more effective than SAQ in A549 cells.

##### Sequential Drug Administration

**Figure 19:**
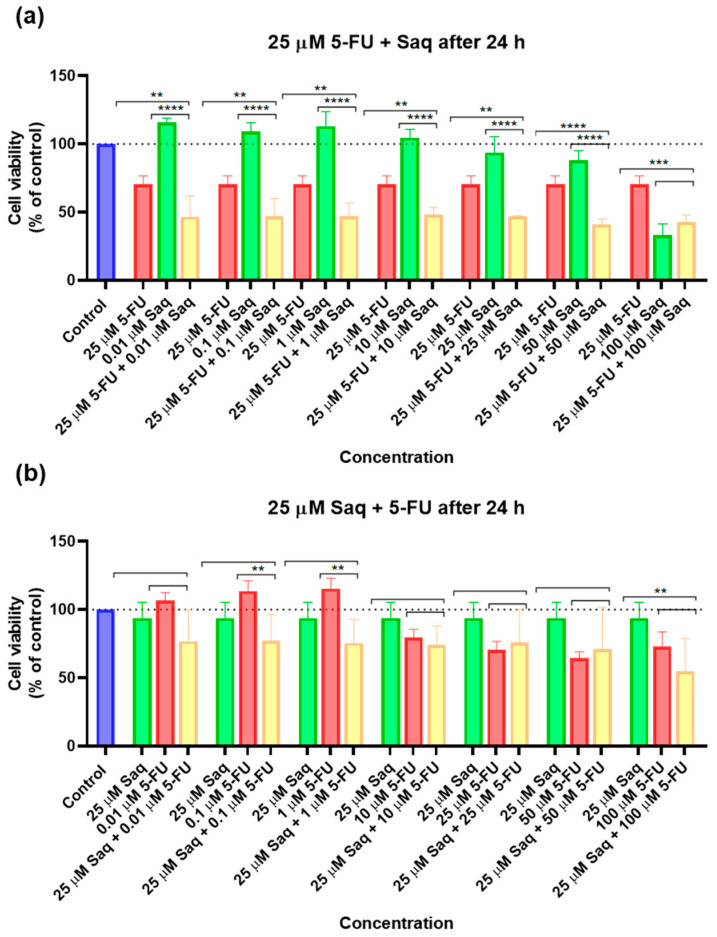
Cytotoxic results of A549 after exposure to drug combinations with the second drug only being added 24 h after the first. (**a**) Combination of 25 μM of 5-FU with increasing concentrations of saquinavir (0.01–100 μM); (**b**) combination of 25 μM of saquinavir with increasing concentrations of 5-FU (0.01–100 μM). Control cells were treated with 0.01% DMSO (vehicle). Cell viability was obtained using the MTT assay after 48 h, and the results are given as the mean ± SEM (*n* = 3). ** statistically significant vs. drug alone at *p* < 0.01; *** statistically significant vs. drug alone at *p* < 0.001; **** statistically significant vs. drug alone at *p* < 0.0001.

**Figure 20:**
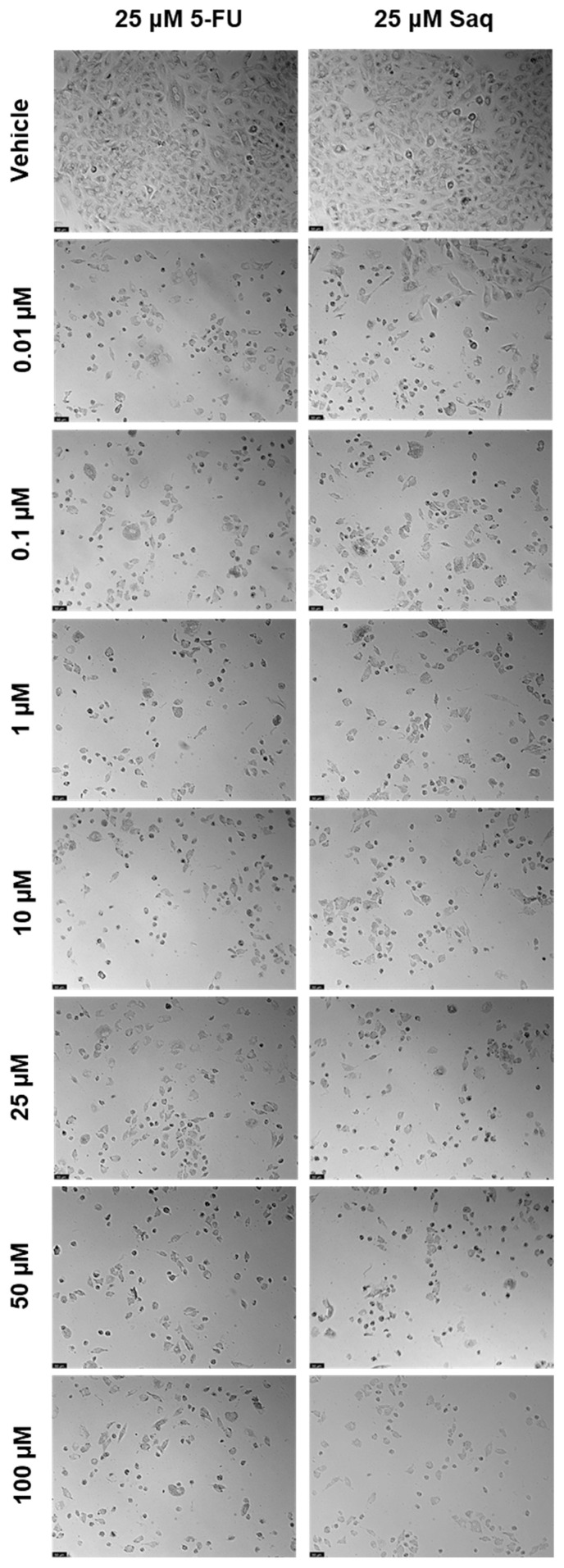
Morphological evaluation of A549 after exposure to combinations of 25 μM of 5-FU with increasing concentrations of saquinavir (0.01–100 μM) or 25 μM of saquinavir with increasing concentrations of 5-FU (0.01–100 μM) for 48 h. The second drug was only added 24 h after the first drug. Control cells were treated with the vehicle (0.01% DMSO). These images are representative of three independent experiments. The scale bar is 200 μM.

The results of cell viability and morphological evaluation of drug combinations where the second drug was added 24 h after the fixed drug are shown in Figure 19 and Figure 20, respectively. In contrast to when drugs are added at the same time, the combinations of 25 μM of 5-FU with increasing concentrations of SAQ 24 h later were more effective in decreasing cell viability than both drugs alone, except for the highest concentration of SAQ (Figure 19a). However, between the combinations, the values are similar despite the increasing SAQ concentrations. For 25 μM of SAQ with delayed increasing concentrations of 5-FU, the results are similar to the ones obtained for the drugs administered at the same time (Figure 19b). Only the combinations with 0.1 and 1 μM of 5-FU were better than 0.1 and 1 μM of 5-FU alone, and the combination of 100 μM of 5-FU was better than 25 μM alone. 

##### Synergism Results

Table 5 demonstrates the results obtained from the synergy studies using CompuSyn. Most of the combinations were shown to be synergistic, except for the combination of 25 μM of SAQ with 100 μM of 5-FU both when administrated at the same time or with a 24 h delay. Indeed, in the combination of 25 μM of SAQ with concentrations of 5-FU greater than 10 μM, the higher the concentration of 5-FU was, the lower the synergism was, until it became antagonistic, indicating that there is a threshold for 5-FU that can be combined with 25 μM of SAQ. For values lower than 10 μM the combinations had the most synergy, mainly with a 24 h delay of 5-FU, but they also had a low effect on cellular death. 

In contrast, the combination of 25 μM of 5-FU with various concentrations of SAQ had a higher effect, particularly when SAQ was added 24 h later, with synergetic combinations, even if the synergism was decreased with the increase of SAQ concentration. Taking all the results together, the better combination in A549 cells seems to be 25 μM of 5-FU with 24 h delayed addition of increasing concentrations of SAQ, which is according to the cell viability graphs. 

These results for A549 cells seem to be in direct opposition to the results for PC-3 cells, where the best combination was 25 μM of SAQ with 5-FU after 24 h. The fractional effect of all combinations in PC-3 was also greatly superior to the effect in A549 cells (Table 2), showing that PC-3 cells seem to be the most sensitive to SAQ and 5-FU.

## 3. Discussion

SAQ is a competitive inhibitor of the HIV-protease, and its pharmacokinetic profile is characterized by extensive metabolization by CYP3A4 of the cytochrome P450 in the liver [20]. This drug has been thoroughly studied for drug repurposing in multiple types of cancer. Several potential modes of action have been proposed, namely: (i) inhibition of the 20s and 26s proteasome [17]; (ii) inactivation of the apoptosis inhibitor nuclear factor kappa B (NF-κB) [15]; (iii) inhibition of the phosphatidylinositol 3-kinases-protein kinase B (PI3K-Akt) radiation-resistance inducer pathway [18]; and (iv) inhibition of angiogenesis and cell invasion [11]. In this study we aimed to evaluate the effect SAQ has on prostate cancer PC-3 cells and lung carcinoma A549 cells, focusing on this drug alone and in combination with the anticancer drug 5-FU. A combination of repurposed drugs with oncologic drugs is a strategy that allows a potentially greater effect than the drugs would be able to achieve when administrated alone, while also using lower dosages.

Cell viability after treatment with increasing SAQ concentrations was obtained using the MTT assay. For PC-3 cells, this drug demonstrated a marked concentration-dependent decrease in cell viability and we were able to obtain IC_50_ values for all times tested. When compared with the in vitro IC_50_ of SAQ in HIV treatment, the values we obtained were higher, both in comparison with those obtained with human serum (37.7 ± 5 nM) and without human serum (1–30 nM) [21]. However, when compared with the only other IC_50_ we could find in the literature for PC-3 cells, which was 37.5 μM for 24 h, the value we obtained for the same time point was lower (20.98 μM). The difference could be attributed to different cell viability assays (MTT vs. crystal violet) [22]. The values obtained were also similar across time points, contrary to the decrease of IC_50_ with a longer exposure time than would normally be expected, which leads to the conclusion that SAQ exerts its effect quickly after administration in PC-3 cells, which is ideal since this drug is quickly metabolized and has a fast systemic clearance [23]. Meanwhile, 5-FU did not have as strong an effect as SAQ had, and we could not achieve any real IC_50_ results. Furthermore, 5-FU is an antimetabolite analog of uracil drugs that disrupts RNA synthesis and inhibits the thymidylate synthase enzyme, causing DNA damage [24]. This drug has shown the ability to radiosensitize prostate cancer cells before [25]. Furthermore, 5-FU is a drug that is potentiated when there is a reduction of folates, and prostate cancer cells overexpress a prostate-specific membrane antigen (PSMA), which is a folate hydrolase [26]. These two indications made us choose 5-FU as a reference cancer drug to test in this study, and even if we did not obtain an IC_50_, significant cytotoxicity to prostate cancer cells was observed, which prompted us to continue using this drug in the combination studies. Cytotoxicity could be even greater if radiation was also applied to these cells, due to the radiosensitization ability mentioned previously. 

In A549 cells, SAQ did not have as strong an effect as it had in PC-3 cells, with 24 h not being enough to be able to obtain an IC_50_ in this cell line. The values that could be obtained for a prolonged time were more than double those for PC-3 and are higher than those obtained in other studies for the same lung cancer cell line, which was around 25 μM [27]. In opposition, 5-FU presented a marked effect on the cell viability of lung cancer cells, with low IC_50_ values compared with SAQ and even with SAQ and 5-FU in prostate cancer cells. This is interesting since a previous study showed that A549 cells are somewhat resistant to 5-FU treatment, with an IC_50_ of around 70 μM for 72 h [28]. 

After the IC_50_ of SAQ was determined and we ascertained that 5-FU had cytotoxic activity in PC-3 cells, we decided to perform combination studies of these two drugs, using the closest concentration we had to that of the IC_50_ of SAQ (25 μM), and, while we could not determine the IC_50_ of 5-FU, and since the concentration of 25 μM had an effect close to 50% at 48 h as well (Figure 1b), we decided to test the same concentrations for both drugs. As expected, fixating the concentration of 5-FU and increasing SAQ proved to have more influence in the combination than when the SAQ concentration was fixed, which shows that most of the cytotoxicity was due to SAQ, this being the more effective drug. This was true both when the drugs were added at the same time and when they were added 24 h apart, with results being close to each other but slightly more effective when SAQ had more time to act on cells. This also proves that SAQ does indeed have most of its activity shortly after being administered. 

While varying the SAQ concentration offered the combination groups the most variation, the combination with better synergy and greater decrease of cell viability was when the IC_50_ of SAQ was used in combination with increasing concentrations of 5-FU, particularly when the drugs were added 24 h apart. Generally, simultaneous administration of drug combinations tends to be more effective than sequential administration, but this is usually followed by higher side effects risk, despite sequential combination allowing for the use of higher concentrations and for a longer therapy time [29]. However, there have been studies that demonstrated that, much like our results, administering drugs once followed by another 24 h later was more potent than simultaneous therapy [30]. In our study, this could be a result of 5-FU in some way potentiating later SAQ effects, while in earlier stages when given at the same time could compete with SAQ. It has been shown before that 5-FU downregulates the multi-drug resistance transporter protein P-glycoprotein (P-gp) [31], and it is well known that SAQ is a substrate of this transporter and that its presence decreases the intracellular accumulation of this drug [32]. Therefore, it could be that 5-FU’s downregulation of P-gp could increase intracellular accumulation and potentiate SAQ’s activity [33]. 

Meanwhile, for A549 cells, the combination of SAQ and 5-FU was not very effective overall. The better option was 25 μM of 5-FU with increasing concentrations of SAQ administered at an interval of 24 h, which is curious since in the single drug studies SAQ did not have a strong effect in this cell line in 24 h. This could indicate that, in these cells, it is the 5-FU’s prolonged effect that is potentiated by its combination with SAQ. On the other hand, the combination of higher concentrations of SAQ and 5-FU seems to be antagonistic, particularly with later administration, which could indicate a threshold of concentrations for these drugs when used in combination. It is known that 5-FU is degraded by dihydropyrimidine dehydrogenase (DPD), an enzyme related to pyrimidine degradation, which makes DPD levels important for 5-FU activity [34]. Indeed, it has been shown that patients of non-small cell lung cancer with high DPD expression treated with 5-FU have a decreased 5-year survival rate [35]. Therefore, limiting this enzyme is important for 5-FU treatment success. It was discovered previously that some antivirals, such as brivudine, can inhibit DPD, impeding 5-FU degradation and potentiating its effects [36]. Although this is a different antiviral drug, this kind of mechanism could be similar for saquinavir, and the addition of this drug after 24 h could impede the long-term degradation of 5-FU, allowing it to act for a longer time in these lung cancer cells, having a stronger cytotoxic effect.

## 4. Materials and Methods

### 4.1. Cell Culture and Reagents

PC-3 human prostate carcinoma and A549 lung carcinoma epithelial cell lines were used to assess the toxicity of SAQ and 5-FU. These cell lines were acquired from the American Type Culture Collection (ATCC, Manassas, VA, USA) and the drugs were obtained from Sigma-Aldrich (Merck KGaA, Darmstadt, Germany). Cells were maintained at 37 °C and 5% CO_2_ in Dulbeco’s modified Eagle’s medium (DMEM) supplemented with 1% penicillin-streptomycin solution and 10% fetal bovine serum (FBS), with all reagents having been obtained from Millipore Sigma (Merck KGaA, Darmstadt, Germany). For the maintenance, trypsinization of confluent cells was performed using a solution of 0.25% trypsin-EDTA (Gibco; Thermo Fisher Scientific, Inc., Waltham, MA, USA), followed by subculture in a new DMEM medium, with a renewal of it every 96 h. For the experiments, 96-well plates were seeded with a density of 5000 PC-3 cells per well (passages 21–33) and 8000 A549 cells per well, which were left to adhere overnight. 

### 4.2. Drug Treatment

Cytotoxicity of SAQ and 5-FU were evaluated alone after 24 h, 48 h, and 72 h, using concentrations of 0.01, 0.1, 1, 10, 25, 50, and 100 μM. 

For the combination studies, the previously mentioned concentrations were tested in combination with 25 μM of SAQ or 5-FU, with the drugs being added to the cells at the same time or with the second drug (that corresponds with the one with increasing concentrations) being added 24 h after the fixed concentration drug. Results were obtained after 48 h.

In all cases, the control cells were treated with 0.1% of the vehicle in which the drugs were dissolved, which in this case was dimethyl sulfoxide (DMSO).

### 4.3. Morphological Analysis

After the determined time of incubation of the drugs, cell morphology was assessed using a Leica DMI 6000B microscope equipped with a Leica DFC350 FX camera (Leica Microsystems, Wetzlar, Germany). Images obtained were then analyzed using Leica LAS X imaging software (v3.7.4) (Leica Microsystems, Wetzlar, Germany).

### 4.4. MTT Assay

The toxicity of the tested drugs was determined via the MTT (thiazolyl blue tetrazolium bromide) colorimetric assay. After the chosen time, the cell culture medium was aspirated and 100 μL of a solution of 0.5 mg/mL of MTT in PBS (Sigma-Aldrich; Merck KGaA, Darmstadt, Germany) was added. The cells were incubated with this solution for 2 h at 37 °C and 5% CO_2_ in total darkness, after which the MTT solution was aspirated, and the purple formazan crystals formed were solubilized with 100 μM of DMSO. The absorbance was read at 570 nm by using an automated microplate reader (Tecan Infinite M200, Tecan Group Ltd., Männedorf, Switzerland), with cell viability being calculated by comparing the absorbance reads of the experimental groups with the control group.

### 4.5. Statistical Analysis

To create the cell viability graphs, GraphPad Prism 9 software (GraphPad Software Inc., San Diego, CA, USA) was used, with results being shown as the cell viability mean ± SEM. The one-way ANOVA tests by Dunnett’s multiple comparisons between control and experimental groups of drugs alone were used. For combination studies, a two-way ANOVA was performed, and the viability results of the combinations were compared with the viability results of each drug at the respective concentration. Statistical significance was set at *p* values < 0.05.

To produce the dose-response curves, the viability results were first normalized with the viability of the control group and plotted with the logarithmized drug concentrations, using a non-linear regression test.

### 4.6. Synergism Study

To quantify the interactions between SAQ and 5-FU in combination, CompuSyn software (ComboSyn, Inc., New York, NY, USA) was used. This program applies the unified theory of Chou and Talalay [37] to obtain the combination index (CI) for each combination, choosing the mutually exclusive model, under the assumption that drugs had different modes of action. SAQ and 5-FU were combined in a non-constant ratio (*n* = 3) and results of CI inferior to one indicate synergism, of one indicate additivity, and a CI superior to one indicates that the drugs had an antagonistic effect. 

## 5. Conclusions

Taken together, these results demonstrate that SAQ had a strong effect on PC-3 cells with a low concentration and could be a new drug to repurpose for prostate cancer treatment. Furthermore, 5-FU could also be utilized, although it had a weaker effect. The combination of these two drugs is also very promising when using a concentration close to the IC_50_ of SAQ and even small concentrations of 5-FU, with drugs added in a sequential form. This is important since it decreases the potentially toxic side effects that could arise from the use of either high concentrations or from the simultaneous administration of two drugs, one of which is an anticancer drug and is known for its high toxicity. However, for A549 cells, the use of SAQ was not as effective, with 5-FU being the one that had a stronger impact on cell viability. Even in combination, the overall cytotoxicity was weaker in comparison with PC-3 cells. The high IC_50_ and the weak combination effects indicate that, for lung cancer, SAQ is not a very promising repurposed drug, when compared with prostate cancer.

This work demonstrated a novel study of the combination of SAQ in prostate and lung cancer cells, as well as a new potential therapeutic drug for prostate cancer. Nonetheless, further investigation is needed to understand the mechanism of action behind the effectivity of the SAQ and 5-FU combination, particularly in prostate cancer, as well as why a sequential combination is better than a simultaneous one. 

## Figures and Tables

**Table 1:** Values of IC_50_ obtained after exposure of PC-3 cells to 5-FU and SAQ for various time points.

Time	5-FU (μM)	SAQ (μM)
24 h	-	20.98
48 h	-	21.71
72 h	7.939	18.97

**Table 2:** Effect and CI values of 5-FU and SAQ combinations simultaneously or with the second drug being added after 24 h. CI < 1 represents synergism, CI = 1 represents additivity, and CI > 1 represents antagonism. The fractional effect shows the degree of cell death, ranging from 0 to 1, with 0 being no cellular death and 1 being total cellular death.

Dose 5-FU (µM)	Dose SAQ (µM)	Fractional Effect (Fa)		CI	
		Drugs at the Same Time	Second Drug after 24 h	Drugs at the Same Time	Second Drug after 24 h
25	0.01	0.49693	0.46170	0.33368	0.34836
	0.1	0.48003	0.45998	0.34553	0.35483
	1	0.46223	0.50216	0.41007	0.37695
	10	0.48438	0.52783	0.86473	0.69645
	25	0.73474	0.55407	0.40504	1.07532
	50	0.89764	0.73382	0.20656	0.56943
	100	0.89802	0.87249	0.24013	0.29682
0.01	25	0.77765	0.85765	0.10247	0.03567
0.1		0.76973	0.85007	0.11271	0.04084
1		0.76928	0.84064	0.12156	0.05411
10		0.75946	0.85822	0.21870	0.11233
25		0.78817	0.85686	0.31394	0.22933
50		0.77396	0.86172	0.56437	0.41524
100		0.79666	0.86666	0.96097	0.78490

**Table 3:** Values of IC_50_ were obtained after exposure of A549 cells to 5-FU and SAQ for various time points.

Time	5-FU (μM)	SAQ (μM)
24 h	48.03	-
48 h	8.891	58.10
72 h	6.070	41.04

**Table 4:** IC_50_ values for A549 and PC-3 cells after exposure to 5-FU and SAQ for various time points.

Time	5-FU (μM)		SAQ (μM)	
	PC-3	A549	PC-3	A549
24 h	-	48.03	20.98	-
48 h	-	8.891	21.71	58.10
72 h	7.939	6.070	18.97	41.04

**Table 5:** Effect and CI values of 5-FU and SAQ combinations simultaneously or with the second drug being added after 24 h in A549 cells. CI < 1 represents synergism, CI = 1 represents additivity, and CI > 1 represents antagonism. The fractional effect shows the degree of cell death, ranging from 0 to 1, with 0 being no cellular death and 1 being total cellular death.

Dose 5-FU (μM)	Dose Saq (μM)	Fractional Effect (Fa)		CI	
		Drugs at the Same Time	Second Drug after 24 h	Drugs at the Same Time	Second Drug after 24 h
25	0.01	0.34927	0.53259	0.37859	0.29666
	0.1	0.37333	0.53139	0.36624	0.29731
	1	0.44084	0.52818	0.33653	0.30037
	10	0.41440	0.51788	0.37013	0.32289
	25	0.52470	0.53178	0.35049	0.34721
	50	0.50016	0.59234	0.41478	0.36583
	100	0.59071	0.57037	0.45854	0.47192
0.01	25	0.14231	0.23028	0.10353	0.08302
0.1		0.02529	0.22898	0.21105	0.08490
1		0.05188	0.24309	0.18825	0.09855
10		0.33477	0.25681	0.22279	0.25312
25		0.46224	0.24060	0.38079	0.53012
50		0.36769	0.28678	0.80230	0.90553
100		0.38416	0.45196	1.50517	1.37422

## Data Availability

Not applicable.

## References

[1] Cancer.Net Prostate Cancer: Statistics. https://www.cancer.net/cancer-types/prostate-cancer/statistics.

[2] National Cancer Institute Cancer Stat Facts: Prostate Cancer. https://seer.cancer.gov/statfacts/html/prost.html.

[3] Nguyen-Nielsen M., Borre M. (2016). Diagnostic and Therapeutic Strategies for Prostate Cancer. Semin. Nucl. Med..

[4] Rebbeck T.R. (2017). Prostate Cancer Genetics: Variation by Race, Ethnicity, and Geography. Semin. Radiat. Oncol..

[5] Mao Y., Yang D., He J., Krasna M.J. (2016). Epidemiology of Lung Cancer. Surg. Oncol. Clin. N Am..

[6] Bade B.C., Dela Cruz C.S. (2020). Lung Cancer 2020: Epidemiology, Etiology, and Prevention. Clin. Chest Med..

[7] World Cancer Research Fund International Lung Cancer Statistics. https://www.wcrf.org/cancer-trends/lung-cancer-statistics/.

[8] Pushpakom S., Iorio F., Eyers P.A., Escott K.J., Hopper S., Wells A., Doig A., Guilliams T., Latimer J., McNamee C. (2019). Drug repurposing: Progress, challenges and recommendations. Nat. Rev. Drug Discov..

[9] la Porte C.J. (2009). Saquinavir, the pioneer antiretroviral protease inhibitor. Expert Opin. Drug Metab. Toxicol..

[10] Vella S., Floridia M. (1998). Saquinavir. Clinical pharmacology and efficacy. Clin. Pharm..

[11] Sgadari C., Barillari G., Toschi E., Carlei D., Bacigalupo I., Baccarini S., Palladino C., Leone P., Bugarini R., Malavasi L. (2002). HIV protease inhibitors are potent anti-angiogenic molecules and promote regression of Kaposi sarcoma. Nat. Med..

[12] Gupta A.K., Cerniglia G.J., Mick R., McKenna W.G., Muschel R.J. (2005). HIV protease inhibitors block Akt signaling and radiosensitize tumor cells both in vitro and in vivo. Cancer Res..

[13] Bandiera E., Todeschini P., Romani C., Zanotti L., Erba E., Colmegna B., Bignotti E., Santin A.D., Sartori E., Odicino F.E. (2016). The HIV-protease inhibitor saquinavir reduces proliferation, invasion and clonogenicity in cervical cancer cell lines. Oncol. Lett..

[14] Timeus F., Crescenzio N., Ricotti E., Doria A., Bertin D., Saglio G., Tovo P.A. (2006). The effects of saquinavir on imatinib-resistant chronic myelogenous leukemia cell lines. Haematologica.

[15] Timeus F., Crescenzio N., Doria A., Foglia L., Pagliano S., Ricotti E., Fagioli F., Tovo P.A., Cordero di Montezemolo L. (2012). In vitro anti-neuroblastoma activity of saquinavir and its association with imatinib. Oncol. Rep..

[16] Kraus M., Müller-Ide H., Rückrich T., Bader J., Overkleeft H., Driessen C. (2014). Ritonavir, nelfinavir, saquinavir and lopinavir induce proteotoxic stress in acute myeloid leukemia cells and sensitize them for proteasome inhibitor treatment at low micromolar drug concentrations. Leuk Res..

[17] Pajonk F., Himmelsbach J., Riess K., Sommer A., McBride W.H. (2002). The human immunodeficiency virus (HIV)-1 protease inhibitor saquinavir inhibits proteasome function and causes apoptosis and radiosensitization in non-HIV-associated human cancer cells. Cancer Res..

[18] Plastaras J.P., Vapiwala N., Ahmed M.S., Gudonis D., Cerniglia G.J., Feldman M.D., Frank I., Gupta A.K. (2008). Validation and toxicity of PI3K/Akt pathway inhibition by HIV protease inhibitors in humans. Cancer Biol. Ther..

[19] Wigmore P.M., Mustafa S., El-Beltagy M., Lyons L., Umka J., Bennett G. (2010). Effects of 5-FU. Adv. Exp. Med. Biol..

[20] Fitzsimmons M.E., Collins J.M. (1997). Selective biotransformation of the human immunodeficiency virus protease inhibitor saquinavir by human small-intestinal cytochrome P4503A4: Potential contribution to high first-pass metabolism. Drug Metab. Dispos..

[21] FDA, R.L.I INVIRASE^®^ (Saquinavir Mesylate) CAPSULES and TABLETS. https://www.accessdata.fda.gov/drugsatfda_docs/label/2010/020628s032,021785s009lbl.pdf.

[22] Donia M., Maksimovic-Ivanic D., Mijatovic S., Mojic M., Miljkovic D., Timotijevic G., Fagone P., Caponnetto S., Al-Abed Y., McCubrey J. (2011). In vitro and in vivo anticancer action of Saquinavir-NO, a novel nitric oxide-derivative of the protease inhibitor saquinavir, on hormone resistant prostate cancer cells. Cell Cycle.

[23] EMA Invirase. https://www.ema.europa.eu/en/medicines/human/EPAR/invirase.

[24] Longley D.B., Harkin D.P., Johnston P.G. (2003). 5-fluorouracil: Mechanisms of action and clinical strategies. Nat. Rev. Cancer.

[25] Smalley S.R., Kimler B.F., Evans R.G. (1991). 5-Fluorouracil modulation of radiosensitivity in cultured human carcinoma cells. Int. J. Radiat. Oncol. Biol. Phys..

[26] Manogue C., Ledet E., Guddati A.K., Lewis B., Sartor O. (2018). Extreme Prostate-Specific Antigen Response to Infusional 5-Flourouracil in Castrate-Resistant Prostate Cancer. Oncologist.

[27] Gills J.J., Lopiccolo J., Tsurutani J., Shoemaker R.H., Best C.J., Abu-Asab M.S., Borojerdi J., Warfel N.A., Gardner E.R., Danish M. (2007). Nelfinavir, A lead HIV protease inhibitor, is a broad-spectrum, anticancer agent that induces endoplasmic reticulum stress, autophagy, and apoptosis in vitro and in vivo. Clin. Cancer Res..

[28] Noro R., Miyanaga A., Minegishi Y., Okano T., Seike M., Soeno C., Kataoka K., Matsuda K., Yoshimura A., Gemma A. (2010). Histone deacetylase inhibitor enhances sensitivity of non-small-cell lung cancer cells to 5-FU/S-1 via down-regulation of thymidylate synthase expression and up-regulation of p21(waf1/cip1) expression. Cancer Sci..

[29] Dear R.F., McGeechan K., Jenkins M.C., Barratt A., Tattersall M.H.N., Wilcken N. (2013). Combination versus sequential single agent chemotherapy for metastatic breast cancer. Cochrane Database Syst. Rev..

[30] Eck K.M., Yuan L., Duffy L., Ram P.T., Ayettey S., Chen I., Cohn C.S., Reed J.C., Hill S.M. (1998). A sequential treatment regimen with melatonin and all-trans retinoic acid induces apoptosis in MCF-7 tumour cells. Br. J. Cancer.

[31] Ma Y., Wang Y., Xu Z., Wang Y., Fallon J.K., Liu F. (2017). Extreme low dose of 5-fluorouracil reverses MDR in cancer by sensitizing cancer associated fibroblasts and down-regulating P-gp. PLoS ONE.

[32] Washington C.B., Wiltshire H.R., Man M., Moy T., Harris S.R., Worth E., Weigl P., Liang Z., Hall D., Marriott L. (2000). The disposition of saquinavir in normal and P-glycoprotein deficient mice, rats, and in cultured cells. Drug Metab. Dispos..

[33] Pereira M., Vale N. (2022). Saquinavir: From HIV to COVID-19 and Cancer Treatment. Biomolecules.

[34] Kubota T. (2003). 5-fluorouracil and dihydropyrimidine dehydrogenase. Int. J. Clin. Oncol..

[35] Huang C.L., Yokomise H., Kobayashi S., Fukushima M., Hitomi S., Wada H. (2000). Intratumoral expression of thymidylate synthase and dihydropyrimidine dehydrogenase in non-small cell lung cancer patients treated with 5-FU-based chemotherapy. Int. J. Oncol..

[36] De Clercq E. (2005). Recent highlights in the development of new antiviral drugs. Curr. Opin. Microbiol..

[37] Chou T.C. (2010). Drug combination studies and their synergy quantification using the Chou-Talalay method. Cancer Res..

